# Extraction of brainprint by means of autoencoder with attention mechanism

**DOI:** 10.3389/fncom.2026.1737750

**Published:** 2026-07-01

**Authors:** Muhammed Esad Oztemel, Ömer Muhammet Soysal

**Affiliations:** 1 Division of Electrical and Computer Engineering, Louisiana State University, Baton Rouge, LA, United States; 2 Computer Science, Southeastern Louisiana University, Hammond, LA, United States

**Keywords:** attention mechanism, authentication, autoencoder, biometric, brainprint, EEG, feature representation, identification

## Abstract

Electroencephalography (EEG) signals provide a unique opportunity for personal identification due to their inherent permanence and uniqueness. However, EEG data are complex, multidimensional, non-stationary, and time-dependent, making feature extraction and classification particularly challenging. This study proposes a framework by integrating autoencoder-based feature extraction with self-attention mechanism for EEG-based personal identification utilizing the proposed EEG data cube spatio-temporal stream representation. Three types of stimuli were applied: auditory, cognitive, and resting state. EEG data were collected in-house from college students in two sessions separated by 10 days. A longitudinal design was employed, with Session-1 data used for training and Session-2 data for testing, ensuring robustness against inter-session variability. Extracted features were subsequently classified using Support Vector Machines (SVM), Artificial Neural Networks (ANN), k-Nearest Neighbors (KNN), and Random Forests (RF). SVM achieved accuracy scores 97.62% when identifying two subject, 82.54% when identifying five subjects and 62.90% when identifying seven subjects using the resting state neural patterns. This research highlights the feasibility of using deep learning networks strengthened with a self-attention mechanism for advancing biometric identification through EEG signals.

## 1 Introduction

Research on secure personal identification has been intensively ongoing (Yusop et al., 2025). With the advancement of technologies and information systems, secure access and accurate identity verification have become increasingly important, leading to the development of various security applications. Biometric signals are frequently used in personal identification authorization processes due to their person-specific characteristics; methods such as fingerprint (Karsharma et al., 2024), voice (Patil et al., 2024), and face recognition (Amirgaliyev et al., 2025) have been widely applied for a long time. However, there are also countermeasures capable of bypassing these systems (Fidas and Lyras, 2023). EEG signals, compared to traditional biometric data, possess a more complex structure, making them a more secure option for personal identification (Chan et al., 2018). Nevertheless, due to the inherent complexity of EEG data, performing this task remains a challenging process.

A fundamental distinction in brain-based security systems is the difference between identification and verification, both of which serve distinct operational goals. Identification is the process of determining an individual’s identity by searching a large database for a matching EEG signature, a task often complicated by the increasing probability of false matches as the user base grows (DelPozo-Banos et al., 2014, 2015; Oikonomou, 2025). Conversely, verification seeks to confirm a user identity claim by comparing their live EEG data against a single stored template (Maiorana, 2021). While identification is frequently used in forensic or surveillance contexts, verification is the standard for secure access control in high-security environments (Gui et al., 2019).

EEG signals are simultaneously recorded from multiple locations on the scalp. Depending on the type of stimulus, different regions of the brain generate distinct responses (Genon et al., 2018). Each sensor captures the electrical activity from a specific scalp location; therefore, several studies have focused on analyzing brain responses using data from a single sensor or a selected group of sensors (Abdullah I and Islam, 2022). The topomap of EEG signals provides a spatial visualization of the brain’s overall activity at a specific time point during data collection (Li et al., 2025). Unlike local analyses approaches that focuses on individual channels, topomaps enable the investigation of global spatial brain dynamics. Moreover, concatenating consecutive topomaps results in three-dimensional (3D) EEG data, which enables researchers to observe spatio-temporal patterns of brain activity.

Feature extraction from EEG data is a crucial step in machine learning tasks such as classification (Lotte et al., 2018), prediction (Craik et al., 2019), and identification (Maiorana et al., 2015). Traditional feature extraction techniques, including the Fourier Transform, Power Spectral Density (PSD), Wavelet Transform, and Auto-Regressive (AR) models, have been widely applied to EEG signals. However, (Al-Fahoum and Al-Fraihat, 2014) highlighted several limitations associated with these conventional methods.

In contrast, deep learning algorithms can automatically learn meaningful representations from raw data, eliminating the need for handcrafted feature design. These methods have proven highly effective in a wide range of EEG-related applications, including emotion recognition (Jafari et al., 2023), neurological disorder classification (Lima et al., 2022), brain–computer interface (BCI) development (Tangermann et al., 2012), and personal identification (Becerra et al., 2025). Convolutional Neural Networks (CNNs) are particularly effective at capturing spatial features (Li et al., 2021), while Recurrent Neural Networks (RNNs) are used for modeling temporal dependencies (Salehinejad et al., 2018). Both CNNs and RNNs, either used independently or in hybrid architectures, have demonstrated strong performance in EEG-based biometric research for comprehensive feature extraction (Alzahab et al., 2021).

Autoencoders (AEs) represent another powerful approach for unsupervised feature extraction. An AE encodes input data into a lower-dimensional latent representation and then decodes it to reconstruct the original input as accurately as possible. This mechanism has been extensively applied in EEG research for learning robust and discriminative feature representations (Vallabhaneni et al., 2021).

On the other hand, attention mechanisms determine the relative importance of each feature component with respect to others, allowing the model to focus on the most relevant information. Attention-based approaches have been increasingly applied to EEG signals (Keutayeva and Abibullaev, 2024). In particular, self-attention mechanisms are employed to transform 3D data into an attention-aware representation. This strategy enables the model to emphasize the most informative regions of the data while suppressing less relevant parts, thereby enhancing the overall feature extraction process.

Longitudinal analysis is a challenging task in all EEG related research. Mental state of the participant, type of task or data collection protocol. The BED dataset was introduced to point importance of longitudinal analysis framework in EEG based personal identification (Arnau-González et al., 2021). Furthermore, (Koelsch, 2014) demonstrate overestimation when relying only within-session data in EEG based biometric study.

In this study, we propose an EEG-based personal identification framework that employs a 3D CNN-based autoencoder (AE) integrated with an attention layer for latent feature extraction. The extracted features are then utilized by machine learning models, including Artificial Neural Network (ANN), k-Nearest Neighbor (KNN), Support Vector Machine (SVM), and Random Forest (RF), to perform subject identification. The proposed system was evaluated with datasets containing 7, 5, and 2 subjects. Initially, the models were trained and tested on a dataset from all 7 subjects. Subsequently, a two-subject experiment was conducted to assess subject identifiability. Based on these results, two subjects with lower identifiability were excluded, and the model was regenerated using data from the remaining 5 subjects.

The remainder of this paper is organized as follows: Section 2 reviews related work; Section 3 describes the proposed attention based autoencoder framework; Section 4 outlines the experimental setup; Section 5 presents the results and evaluation metrics; and Section 6 concludes with discussions and future research directions.

## 2 Literature review

For more than 20 years, researchers have actively studied the use of EEG signals for identifying individuals (Fidas and Lyras, 2023). Multiple approaches for gathering EEG data have been created for biometric research purposes. These methods include capturing brain activity during rest, measuring brain responses to external triggers such as sounds, and recording activity while participants perform cognitive tasks (Yang and Deravi, 2017). During resting-state procedures, subjects remain relaxed without engaging in mental or emotional processes (Valizadeh et al., 2019), while stimulus-based and task-based methods require participants to complete specific activities that prompt characteristic brain responses (Kaur et al., 2016).

Recent methodological trends have moved toward hybrid frameworks that combine spatial filtering with deep learning to overcome the non-stationarity of EEG signals. For instance, the use of Common Spatial Patterns (CSP) allows for the design of personalized spatial filters that emphasize subject-specific discriminative characteristics (Oikonomou, 2023). These spatial features are increasingly being integrated into deep learning architectures, such as Siamese Convolutional Neural Networks (CNNs), which learn task-independent deep representations for verification (Maiorana, 2021). Additionally, the application of Sparse Representation Classification (SRC) has emerged as a powerful tool for both identification and verification, utilizing dictionary-based learning to achieve high accuracy even with limited data samples (Oikonomou, 2025).

DelPozo-Banos et al. (2015) examined the use of EEG signals for biometric identification by comparing recordings taken during rest with those captured while participants responded to visual and auditory stimuli. They employed Continuous Wavelet Transform (CWT) to derive time-frequency characteristics from five distinct frequency ranges: delta (0.5–4 Hz), theta (4–8 Hz), alpha (8–13 Hz), beta (13–30 Hz), and gamma (30–50 Hz). The study demonstrated that identification accuracy significantly decreased when training and testing data came from different sessions, falling to roughly 72–85% compared to nearly 99% accuracy achieved using data from a single session. These outcomes underscore the benefits of using stimulus-triggered EEG for identification purposes, though they also highlight the ongoing difficulty of achieving stable performance across different time periods.

Conventional feature extraction methods have various limitations, which has driven researchers to explore more effective feature representation techniques. Soysal et al. (2025) evaluated how different machine learning algorithms performed when using statistical features versus features generated through an autoencoder (AE) for identifying individuals. The findings showed that features obtained through the AE produced better classification results than traditional statistical features. In another study, Bandana Das et al. (2024) utilized an autoencoder to derive latent feature representations from EEG data for person identification purposes. These extracted features were then fed into convolutional neural network (CNN) and long short-term memory (LSTM) models to identify subjects. Similarly, Oztemel and Soysal (2025) used domain adaptive autoencoder for extracting latent features from EEG signals for personal identification. Zhang et al. (2018) developed an autoencoder framework combined with an attention mechanism to process Delta-band EEG recordings from both their proprietary dataset and the publicly accessible EEG Motor Movement/Imagery Dataset (EEGMMIDB). The resulting deep features were subsequently classified using an extreme gradient boosting (XGBoost) algorithm to accomplish subject identification.

Attention mechanisms have rapidly become an important component in EEG modelling because they enable networks to emphasize strong feature components during the learning process. Attention-based modules have been shown to improve performance in many EEG tasks such as emotion recognition and cognitive-state decoding by enhancing discriminative temporal–spatial representations (Ma et al., 2024). Transformer and self-attention architectures in particular have been adapted for EEG decoding to capture long-range temporal dependencies and inter-channel relationships that recurrent or purely convolutional models struggle with; several studies and recent reviews report strong gains from Transformer-style encoders on tasks from motor-imagery to auditory attention decoding (Young-Eun and Seo-Hyun, 2022).

Separate lines of work have also explored autoencoders and variational autoencoders (VAEs) for learning compact, task-agnostic EEG representations useful in downstream tasks, including subject identification and clinical/state classification. These works demonstrate that AE-derived latent features often outperform handcrafted statistical features when used with downstream classifiers (Bandana Das et al., 2024). More recent generative/hybrid models have begun to merge attention-like modules with VAE/autoencoder architectures to improve representation quality for cognitive-state modelling and generative tasks, suggesting attention can aid unsupervised representation learning in EEG (Hu and Guan, 2025).

However, when the literature is examined specifically for EEG based biometric identification, the explicit combination of attention-equipped autoencoders remains relatively sparse. While attention mechanisms and Transformers are well represented across emotion recognition and decoding studies, and autoencoders have been applied to EEG biometric tasks, there are few documented studies that explicitly design attention-augmented autoencoders and evaluate them for longitudinal EEG biometric identification. This gap indicates a promising research direction: integrating attention mechanism into autoencoder based featured learning and evaluating whether the resulting improve cross-session robustness and subject separability for biometric applications.

## 3 Methods

This section describes the proposed framework for EEG-based subject identification and outlines the steps involved in its implementation. A longitudinal analysis was conducted, where all models were trained with a dataset collected during Session 1 and evaluated using the dataset from Session 2. The framework comprises four main modules: 1. Preprocessing, 2. data cube generation, 3. feature extraction using the Attention-based Multi-level Autoencoder (AMAE), and 4. subject identification using four core classifiers—k-Nearest Neighbors (KNN), Support Vector Machine (SVM), Artificial Neural Network (ANN) and Random Forest (RF). All models in this study were developed through hyperparameter search with Bayesian optimization.

### 3.1 Preprocessing

**Figure 1:**
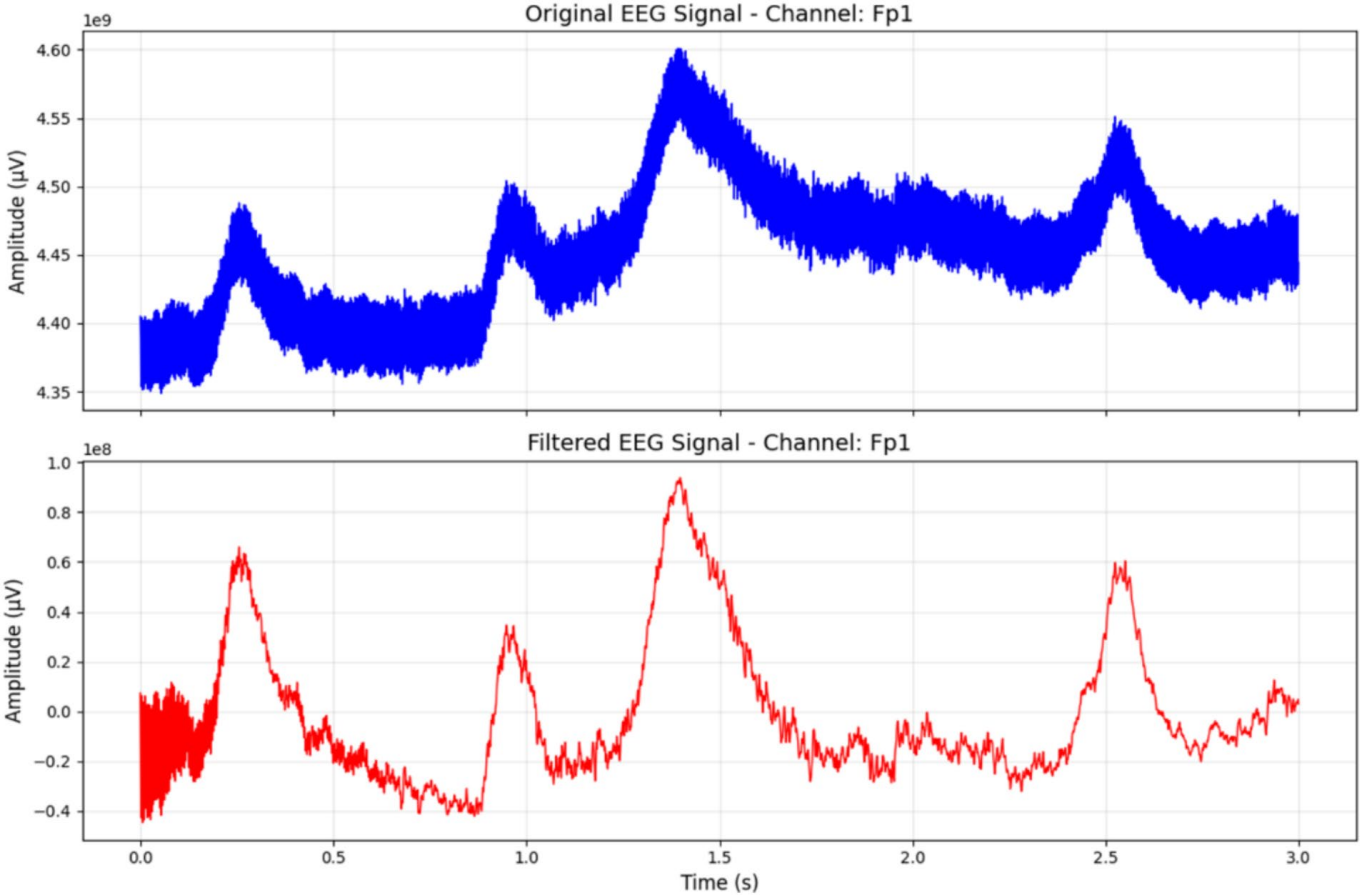
EEG signal preprocessing for channel Fp1. The upper panel shows the raw EEG signal acquired from the Fp1 electrode. The lower panel shows the corresponding preprocessed signal after filtering.

In the preprocessing stage, notch filters were applied to suppress power line interference and its harmonics at 60, 120, 180, and 240 Hz. The notch filter (Dai et al., 2019) selectively attenuates narrowband noise while preserving other frequency components. After line noise removal, a high-pass filter with a 0.5 Hz cutoff was applied to eliminate baseline drifts and low-frequency physiological artifacts. The signals were then band-pass filtered into eight frequency ranges: Delta (1–4 Hz), Theta (4–8 Hz), Alpha (8–13 Hz), Beta (13–32 Hz), Delta–Beta (1–32 Hz), Theta–Beta (4–32 Hz), Gamma (32–125 Hz), and All (>1 Hz). Figure 1 shows an example of the preprocessed EEG signal from a single channel.

### 3.2 Data cube generation

**Figure 2:**
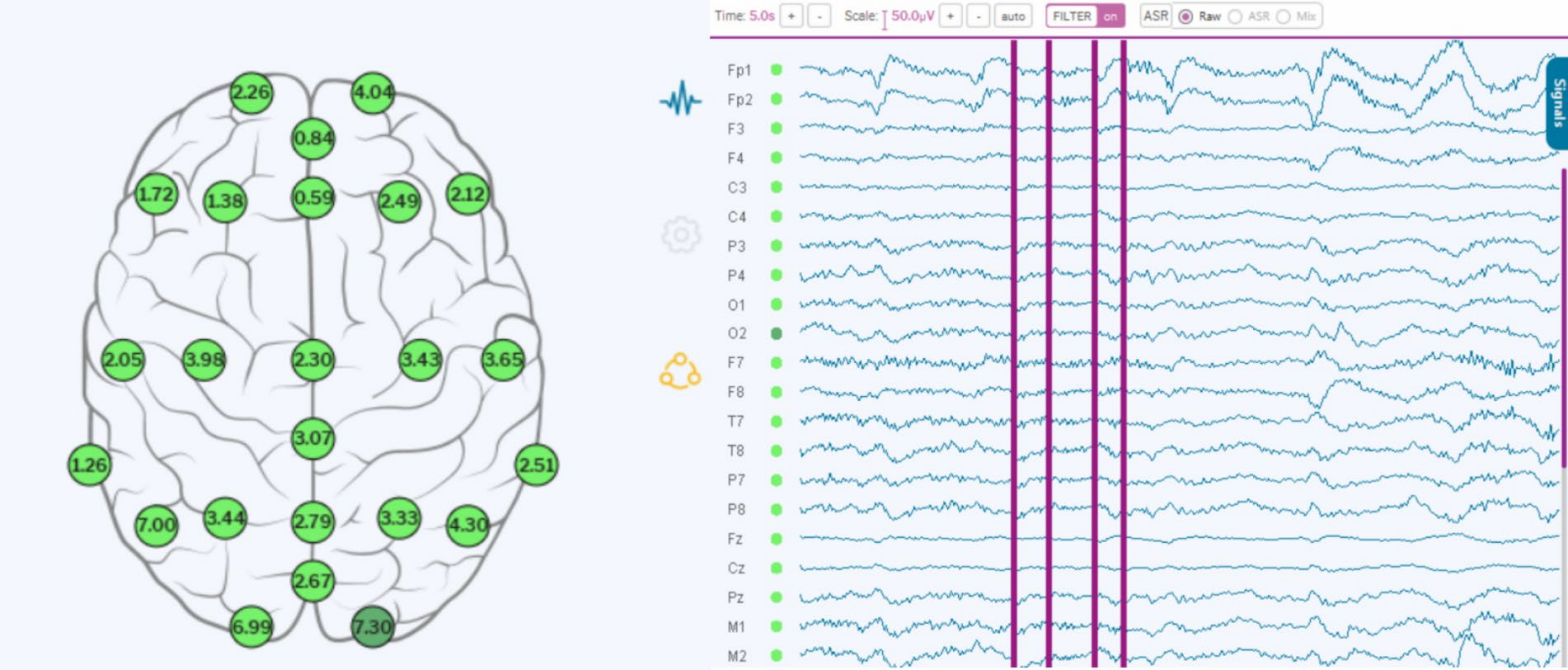
EEG recording session, electrode placement and spatial signal distribution. (Left) Two-dimensional topographic cortical mapping showing the international 10–20 system electrode configuration, with specific localized numerical activation/metric values overlaying each green channel node. (Right) Concurrent multi-channel time-series EEG waveforms mapped from frontal to mastoid channels Fp1 through M2.

EEG signals were recorded using a set of electrodes positioned according to the 10–20 montage system (Oriano, 2019). These electrodes capture the brain’s electrical activity from different scalp locations, providing spatially distributed information across cortical regions. Figure 2 illustrates the electrode placement and an example of EEG recordings obtained while the participant was engaged in specific tasks such as listening to music, performing a cognitive activity, or resting.

Topographic maps (topomaps) were generated by mapping the EEG electrode readings onto a three-dimensional spherical model of the head (Souganttika et al., 2022). The electrode coordinates were subsequently projected onto a two-dimensional plane using an azimuthal projection, which flattens the spherical surface while preserving spatial relationships from the vertex outward. The discrete voltage measurements were then interpolated over a 32 × 32 grid, using bilinear interpolation, producing a smooth distribution over the grid.

**Figure 3:**
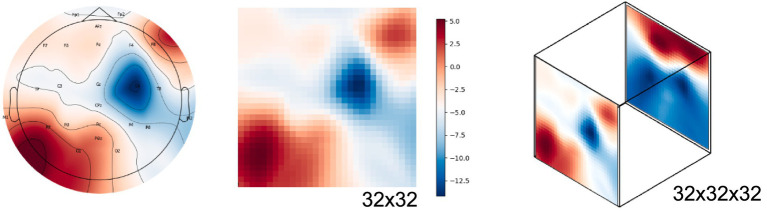
Pipeline for converting topomaps into a spatiotemporal EEG data cube. EEG voltages measured at the electrode positions (left) are interpolated to generate a two-dimensional scalp topographic representation (middle, 32 × 32 resolution). Consecutive topographic maps are then stacked along the temporal axis to form a three-dimensional EEG data cube (right, 32 × 32 × 32), preserving both spatial and temporal characteristics of the EEG activity.

Each EEG signal was recorded over a 3-s interval using a 1 kHz sampling device, resulting in 3000 samples per trial. Minor channel- and trial-level data losses occurred due to intermittent dropouts during acquisition, leaving 2880 valid samples for analysis. To reduce data dimensionality and suppress the artifacts, a median filter W[n,L] with a length *L* given in Equation 1 is applied to the EEG signals f[k] . Experimentally we set *L* = 10, producing a down sampled signal g[n] of a length 288 time points. Eventually, this process produced a three-dimensional EEG data stream $S\in {\mathbb{R}}^{288x32x32}$ . This stream was subsequently divided into nine non-overlapping segments; consequently, 9 (=288/32) data cubes $C\in {\mathbb{R}}^{32x32x32}$ were generated. The dataset had 35, 10, and 10 samples for the resting state, the audio and cognitive tasks, respectively, resulting in 315 (35 samples x 9), 90, 90 (10 samples x 9) data cubes per session. Figure 3 illustrates the process of generating the data cubes from the stack of topomaps.

g[n]=median(f[k]∣k∈W[n,L]) (1)

### 3.3 Autoencoder with attention layer

A three-dimensional convolutional autoencoder (3D-CAE) was employed for automated feature extraction from EEG data cubes $X\;\epsilon \;{\mathbb{R}}^{HxWxT}$ of HxW topomaps stream with temporal length T , as it effectively captures both spatial and temporal dependencies within the signals. Formally, the autoencoder comprises pair of an encoder and decoder functions. The encoder function maps the input tensor $X\;\epsilon \;{\mathbb{R}}^{HxWxT}$ to a latent representation $Z\;\epsilon \;{\mathbb{R}}^{MxMxM}$ , where M denotes the size of a tensor’s dimension. The decoder, then, attempts to reconstruct a data cube $\hat{X}=\mathrm{dec}(\mathrm{enc}(X))$ from Z . The network is optimized by minimizing the reconstruction loss $ℒ(X,\hat{X})$ , defined as the mean squared error between the input X and its reconstruction $\hat{X}$ .

The attention mechanism has been proven to extract distinct patterns in the machine learning field (Soydaner, 2022). We proposed to integrate a self-attention mechanism into the 3D-CAE, allowing the model to emphasize the most informative regions of the feature space and consequently enhancing the discriminative power of the latent representation.

**Figure 4:**
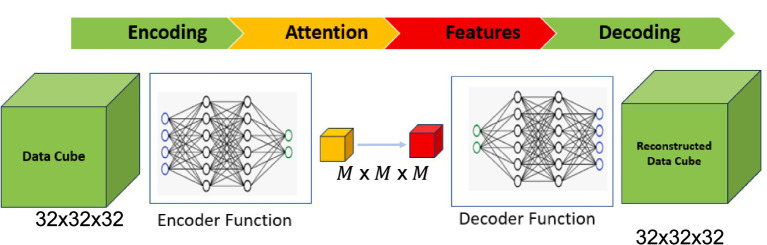
Architectural pipeline of the AMAE for feature extraction. This process flow is divided into three sequential phases: encoding, attention and decoding. Initially, an input 3D data cube of size 32 × 32 × 32 is compressed through the Encoder Function network block. The resulting representations are processed via an Attention mechanism to construct a bottleneck feature representation of dimension *MxMxM*. This optimized feature block is subsequently mapped through the decoder function network to output a reconstructed data cube matching the original input spatial dimensions of 32 × 32 × 32.

To enhance feature extraction from 3D EEG data cubes, an attention mechanism is implemented as a custom layer positioned between the encoder and decoder of a 3D-CNN convolutional autoencoder. The architecture adopts the scaled dot-product attention mechanism, originally proposed by Vaswani et al. (2017) and adapts it for 3D spatial feature processing. The overall structure of the proposed AMAE is illustrated in Figure 4.

**Figure 5:**
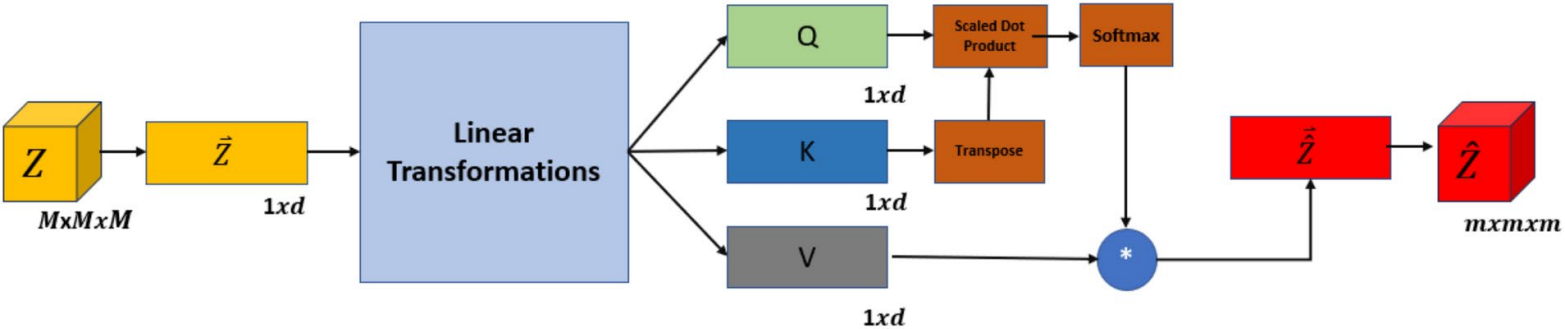
Schematic representation of the scaled dot-product attention mechanism. The architectural workflow maps an initial volumetric tensor into a flatten representation. Through parallel linear transformations *Q*, *K* and *V* vectors are acquired. The matrix operation proceeds via a scaled dot production between *Q* and transpose of *K* which is normalized using a Softmax. The vector *V* is weighed by attention weights to generate attention-based feature vector. Finally, this vector is reshaped back into 3D tensor format.

The attention layer computes three projections vectors query (*Q*), key (*K*), and value (*V*) through linear transformation *d*x*d* matrices $W_{Q},W_{K}$ , and $W_{V}$ applied to the input feature tensor $\vec{Z}\;\epsilon \;{\mathbb{R}}^{1xd}$ . $\vec{Z}$ vector is obtained by flattening the input tensor $Z\;\epsilon \;{\mathbb{R}}^{MxMxM}$ . The linear transformation weights matrices are trained during the learning process. Glorot uniform initialization scheme is used for initialization of these weights to ensure a stable gradient flow during training. The attention mechanism utilizes the *Q* and *K* vectors to generate ‘attention’ weights through the Softmax function operating on the scaled dot-product of *Q* and *K* as given in Equation 2:

$$Q=Z*W_{Q}$$

$$K=Z*W_{K}$$

$$V=Z*W_{V}$$

$$\vec{\hat{Z}}=\text{Attention}(Q,K,V)=\text{Softmax}(\frac{Q.K^{T}}{\surd d})V$$ (2)

where, d denotes the size of the reshaped latent tensor, and √d used for scaling factor to avoid vanishing gradients risk. The dot-product operation measures the similarity of *Q* and *K* vectors. The Softmax function transforms the similarity scores to the range [0,1] assigning a higher weight to the high similarity scores and generating a corresponding weight vector $W_{S}$ . The learning process aims to train transformation matrices to generate highly similar *Q* and *K* vectors. At the final stage of the attention mechanism, the vector V is weighted by $W_{S}$ . Following, $\vec{\hat{Z}}$ is converted back to a data cube to reconstruct $\hat{X}\;\epsilon \;{\mathbb{R}}^{HxWxT}$ . An illustration of this process is presented in Figure 5.

### 3.4 Subject identification

As pointed earlier, a three-dimensional convolutional autoencoder (3D-CAE) is designed to process EEG data cubes, generating compact latent features that retain both spatial and temporal dependencies of the signals. The encoder generates the *N* number of perceptron units at the last layer. The final encoded representation $Z_{M}\in {\mathbb{R}}^{MxMxM}$ is derived from the set of latent features $Z\;\epsilon \;{\mathbb{R}}^{MxMxM}$ which is given in Equation 3 and supplied to the classifiers $\text{model}(Z_{M})$ to calculate the identification score I(X) as given in Equation 4. This two-stage pipeline utilizes the autoencoder’s unsupervised learning ability to produce informative embedding, which in turn improve the performance of downstream supervised classifiers.

$$Z_{M}=\frac{1}{N}\sum_{i=1}^{N}Z_{i}$$ (3)

$$I(X)=\text{model}(Z_{M})$$ (4)

To assess the discriminative capacity and robustness of these learned features, four distinct classification algorithms are implemented: KNN, SVM, ANN, RF. These methods collectively represent diverse learning paradigms, instance-based, margin-based, network, and ensemble-based approaches, respectively. Furthermore, to evaluate cross-session stability in EEG-based subject identification, a longitudinal analysis is performed, where each classifier is trained using data from Session 1 and tested on data collected in a subsequent Session 2.

### 3.5 Parameter search

This research utilizes Keras Tuner with Bayesian optimization to determine the best hyperparameter settings for both autoencoder and classification models. The autoencoder aims to minimize reconstruction error, while the classifiers focus on reducing classification error through optimal hyperparameter selection.

Bayesian optimization uses a model-based approach that efficiently explores the hyperparameter landscape by constructing a probabilistic model and choosing promising options informed by previous results. This method is especially beneficial for deep learning applications where training requires significant computational resources. To assess the framework’s reliability and ability to generalize, a 5-fold cross-validation approach is used for train and validation data split through session-1 data. In each fold, Bayesian optimization runs separately, adjusting model hyperparameters according to the specific training-validation data split. The hyperparameters and their values are provided in Table 1.

**Table 1:** Hyper parameter space.

**Model**	**Parameters**
Autoencoder	Activation Functions: [relu, elu, sigmoid, selu] Optimizers: [adam, adamW] Initializer: [glorot_uniform, glorot_normal, he_uniform, he_normal] Kernel Size: [3 × 3 × 3, 5 × 5 × 3] Number of Layers: [1, 2, 3] Number of Units: [16, 32, 48, 64]
SVM	Kernel: [linear, rbf, poly] Gamma: [scale, auto] C: [min_value = 0.1, max_value = 10, sampling = log]
RF	Number of Estimator: [100, 200, 300] Maximum Depth: [5, 10, 15] Minimum Sample Split: [2, 4, 6, 8, 10] Minimum Sample Leaf: [1, 2, 3, 4, 5] Maximum Features: [sqrt, log2]
KNN	Number of Neighbors: [3, 4, 5] Weights: [uniform, distance] Algorithm: [auto, ball_tree, kd_tree, brute] Leaf Size: [10, 15, 20, 25, 30, 35, 40, 45, 50] Distance: [Manhattan, Euclidean]
ANN	Activation Functions: [relu, elu, sigmoid, selu] Optimizer: [adam, adamW] Initializer: [glorot_uniform, glorot_normal, he_uniform, he_normal] Number of Layers: [1, 2, 3]

## 4 Experimental setup and data

EEG data were collected from seven college students after receiving approval from the university’s Institutional Review Board (IRB). All participants were adults over 18 years old, exhibited normal stress levels, and had no history of neurological conditions. No participant was using medications that could influence neurological activity during data collection. Data acquisition occurred across two sessions in spring 2023, separated by a 10-day interval.

EEG recordings were captured using an mBrain Train amplifier (1 kHz sampling rate) connected to a 24-channel headcap configured according to the international 10–20 electrode placement system, alongside Neuro Behavioral Systems’ Presentation software (version 24.0 07.19.23). Each recording was labelled with a distinct subject identifier: sb106, sb328, sb330, sb381, sb455, sb717, and sb768. Three distinct stimulus conditions were utilized: the resting state, cognitive (inner voicing “evergreen”), and auditory (“canga” sound). During the resting state, participants kept their eyes closed and remained motionless for 3 s without engaging in any activity. The cognitive task required participants to silently repeat the word “evergreen” to themselves for 3 s. In the auditory condition, participants were exposed to a conga drum sound for an equivalent duration. These three stimulus types were analyzed separately to evaluate their effectiveness in person identification. The resting state captures inherent neural patterns, the cognitive task generates internally motivated yet consistent responses, and the auditory task produces externally triggered brain activity. Each session included 35 resting-state, 10 cognitive and 10 auditory EEG recordings, out of 10 trials per session. Each trials include multiple records for resting state stimuli and single record for cognitive and auditory stimuli. The dataset from this study is accessible upon request.

To meet longitudinal study criteria, all training data originated exclusively from Session 1, whereas all testing data came solely from Session 2, maintaining temporal independence between datasets. Model training was conducted using high-performance computing resources at Louisiana State University and the Louisiana Optical Network Initiative (LONI), featuring NVIDIA A100 80GB PCI hardware. All experiments were executed in Python (version 3.12) within the Visual Studio Code (version 1.105) development environment. EEG signal pre-processing and analysis utilized the MNE library, scikit-learn provided machine learning functionalities, TensorFlow and Keras were used for neural network construction, and Keras Tuner facilitated hyperparameter optimization to systematically investigate model configurations and enhance training outcomes.

## 5 Results and discussions

**Figure 6:**
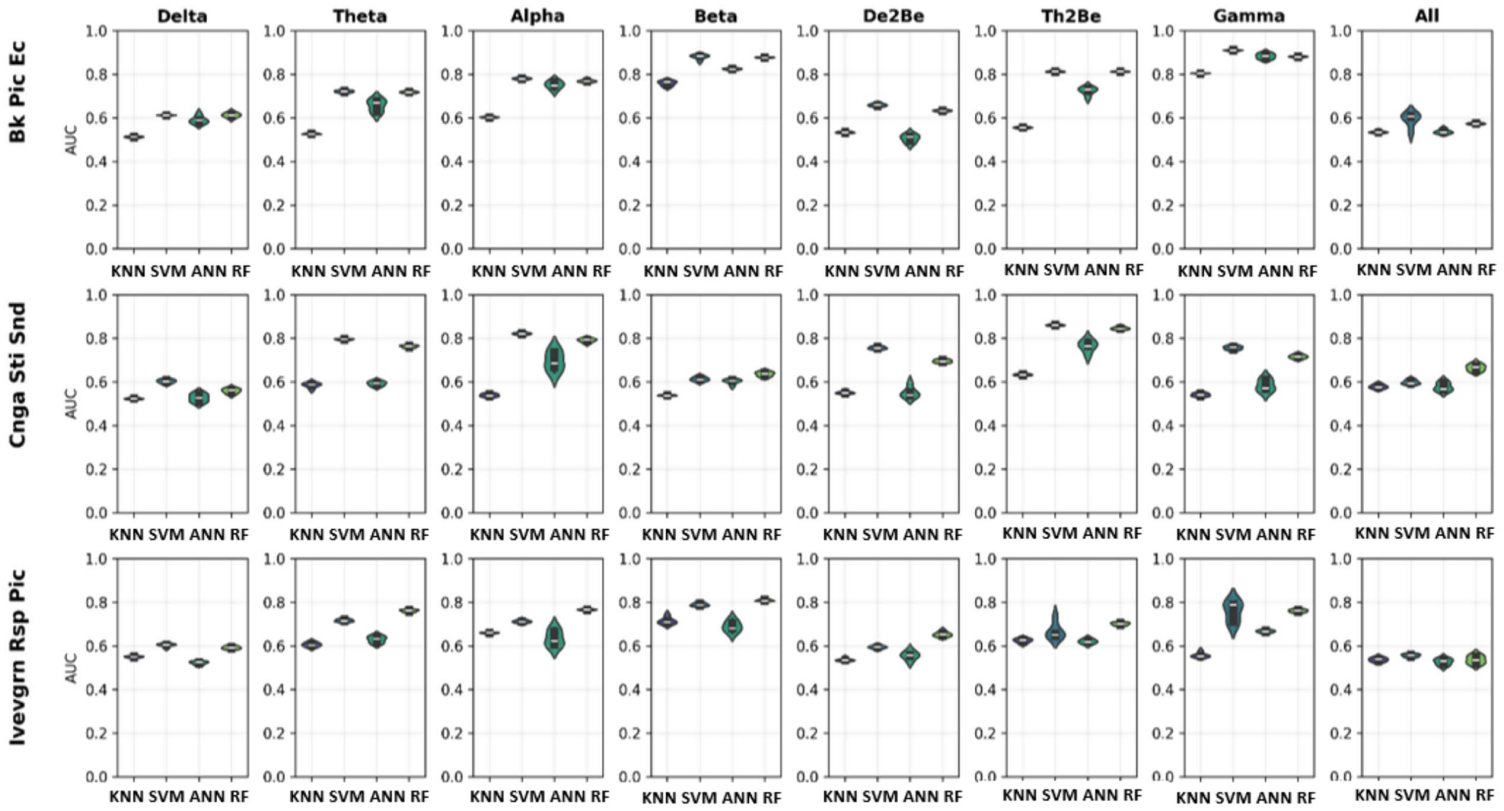
Violin plots for AUC performance of the models across band and stimuli.

Autoencoders were trained independently to extract latent features from spatio-temporal EEG data cubes for eight distinct frequency bands. Subsequently, these extracted features were fed into personal identification models for participant recognition. Several experiments were conducted including effect of distinct EEG bands, the stability of identification using 5-fold cross-validation, the effect of challenging subjects on identification, and subject identifiability. Figure 6 presents the effectiveness of EEG bands obtained by the 5-fold cross-validation in terms of area under curve (AUC) scores. The results demonstrate that the SVM model attained the highest performance with a mean AUC score of 90.94% across 5-fold cross-validation in the Gamma band using resting state EEG data cubes, surpassing all other stimulus types and frequency bands.

Additional experiments were conducted to evaluate SVM-based personal identification in the Gamma band using resting state EEG recordings, specifically examining scenarios involving 2 subjects and 5 subjects. Table 2 provides comprehensive details regarding the network structure and the chosen parameters in Gamma band for resting state EEG records.

**Table 2:** Selected parameters.

**Stimuli–band**	**Autoencoder**	**ANN**	**KNN**	**SVM**	**RF**
**Resting–gamma**	NEs: 250 AF: Relu NLs: 4 KI: Glorot_uniform KS: 3 × 3 × 3 OPT: Adam NUs: 48, 16	NEs: 400 AF: Relu NLs: 2 KI: Glorot_normal OPT: Adam	NNs: 3 WGTs: distance ALGs: auto LS: 25 DSC: Manhattan	KRN: rbf GMA: scale C: 0.98	NESTs: 300 MD: 10 MSS: 6 MSL: 3 MFs: sqrt

NEs: number of epochs, NLs: number of layers, AF: activation function, KI: kernel initializer, KS: kernel size, OPT: optimizer, NUs: number of units, NNs: number of neighbors, WGTs: weights, ALGs: algorithms, LS: leaf size, DSC: distance, KRN: kernel, GMA: Gamma, NESTs: number of estimators, MD: maximum depth, MSS: minimum sample split, MSL: minimum sample leaf and MFs: maximum features.

**Figure 7:**
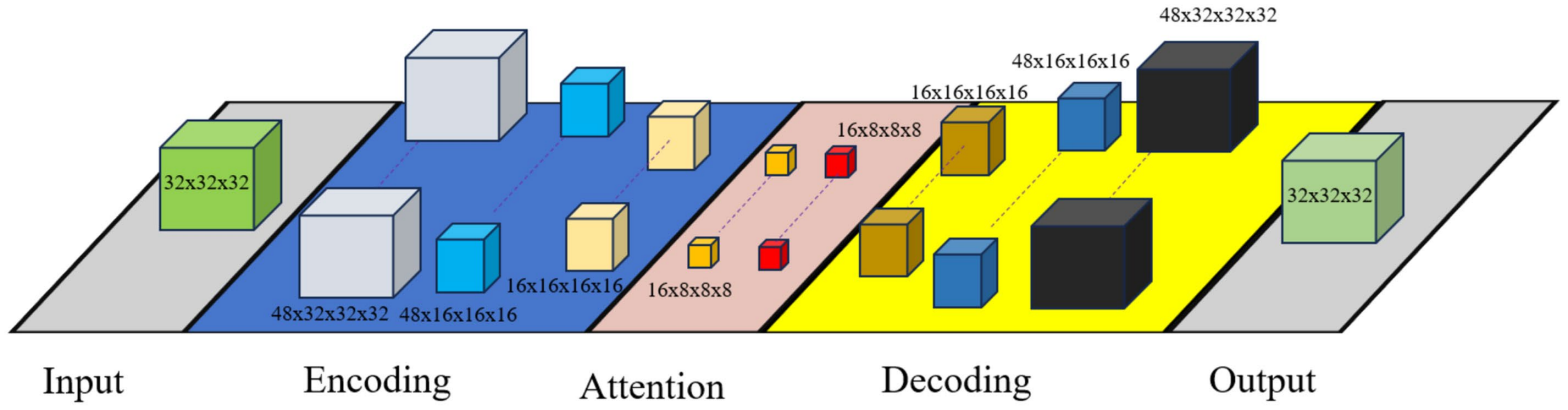
Block diagram of autoencoder for resting state EEG records.

The block diagram of the proposed autoencoder architecture after running the hyperparameter search is provided in Figure 7. EEG data cubes with dimensions of 32 × 32 × 32 are passed through two encoding layers where each has 48 convolution units, resulting in 16 latent feature representations of size 8 × 8 × 8 at the last layer of the encoder. An attention mechanism is then applied to these latent features, and the attention-enhanced representations are subsequently reconstructed back to their original dimensions (32 × 32 × 32).

**Figure 8:**
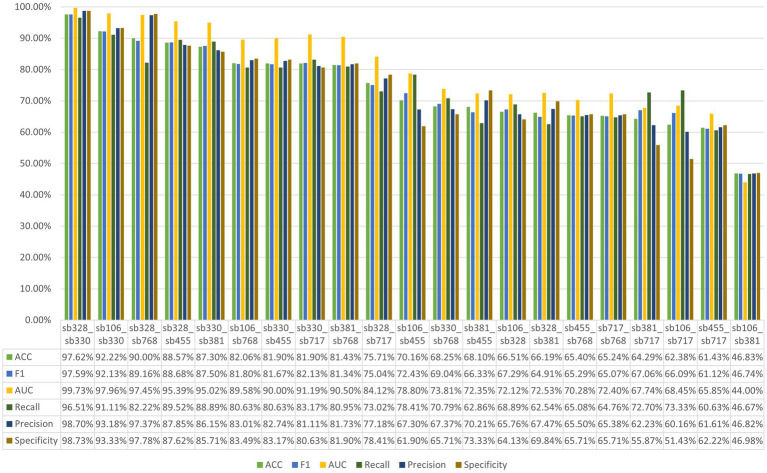
Pairwise identification scores for resting state EEG records in Gamma band.

A pairwise classification was conducted using an SVM-based model to evaluate subject identifiability through the resting state EEG data cubes in Gamma band. All possible combinations among the seven subjects were examined, and the performance of each pairwise model is presented in Figure 8. The highest identification accuracy was obtained for the subject pair sb328 and sb330, achieving an accuracy of 97.62%. Conversely, the lowest performance was observed for the pair sb106 and sb381, with an accuracy of 46.83% and AUC of 44%. The identification performance demonstrates notable variability across subject pairs, as shown in Figure 8. While some subject pairs achieve high accuracy, others show significantly lower performance. This variation can be attributed to differences in the distinctiveness and stability of individual EEG patterns. Subjects with higher identifiability tend to exhibit more consistent and discriminative neural signatures across sessions, whereas subjects with lower performance may present increased intra-subject variability, reduced signal quality, or less separable feature representations. Furthermore, session-related factors and physiological fluctuations may also influence the consistency of EEG signals. These findings underline the importance of considering subject-level variability when evaluating EEG-based biometric systems and motivate the need for more robust and generalized models.

**Figure 9:**
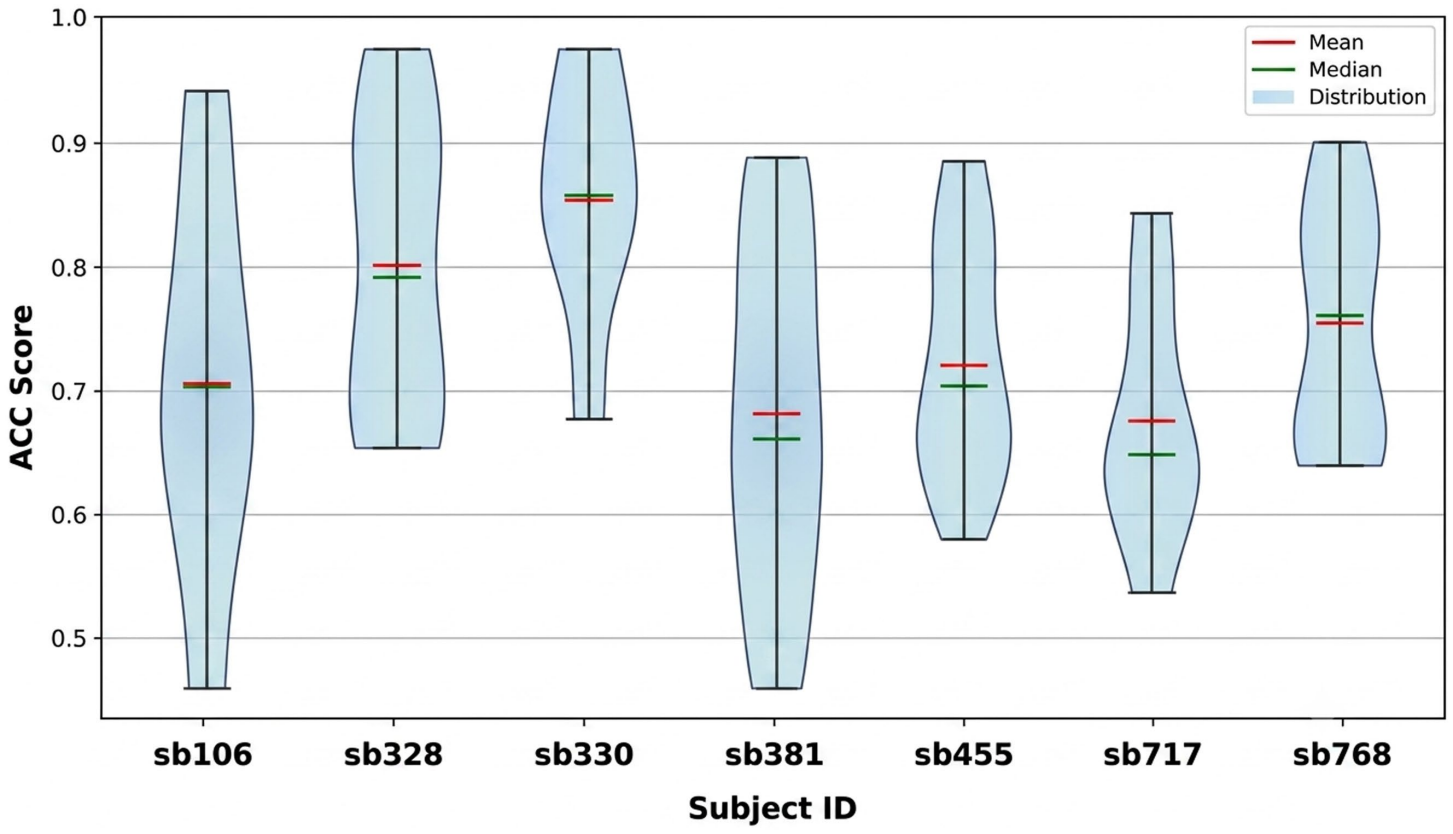
Violin plots for subject identifiability.

Figure 9 presents the degree of intrasubject variability (subject identifiability) in terms of accuracy of identification. As shown in the figure, subjects sb328 and sb330 exhibited highly distinctive brain characteristics for personal identification, whereas sb106 and sb381 demonstrated lower identifiability.

**Figure 10:**
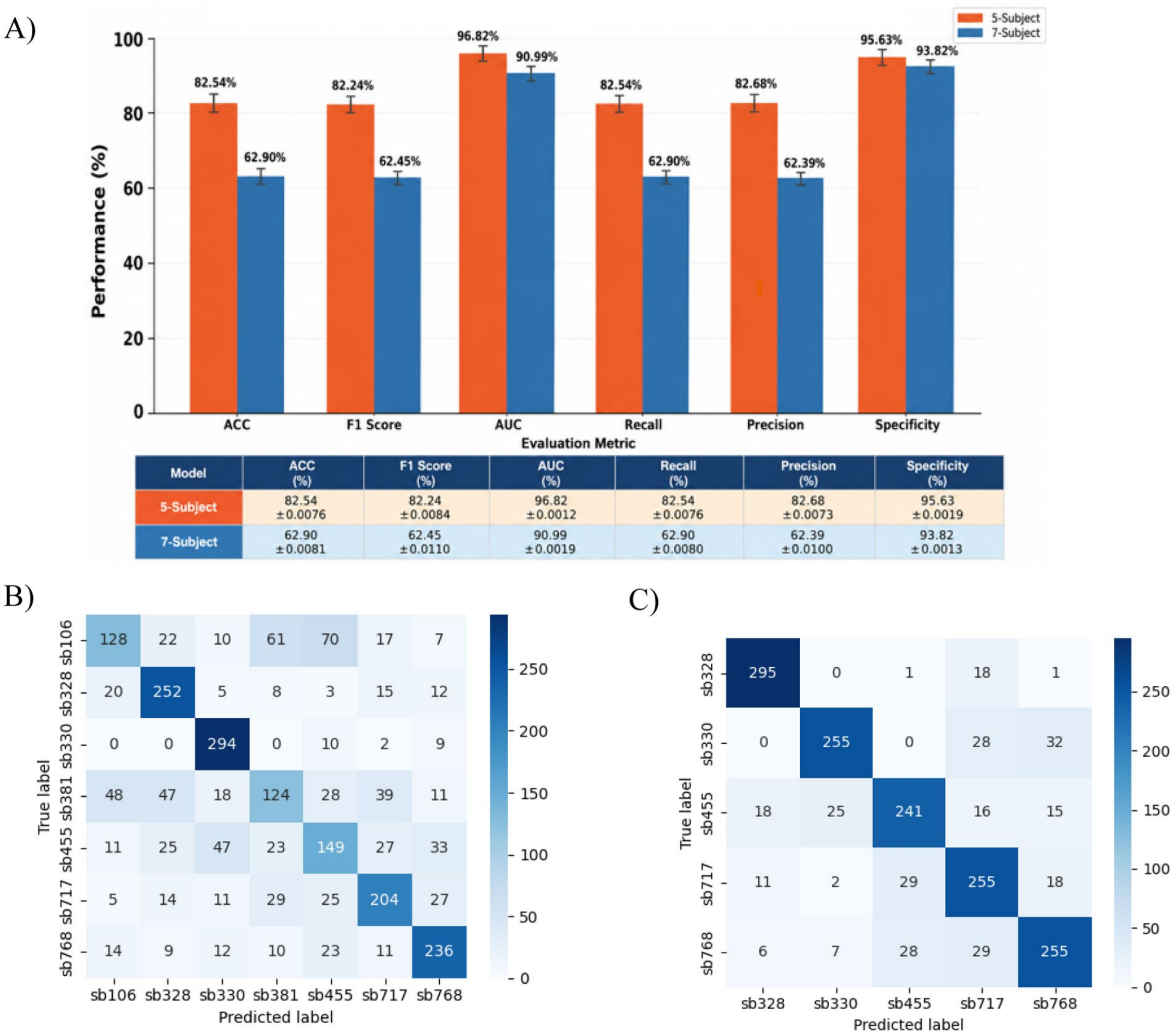
Performance evaluation and comparison of 5-subject and 7-subject experiments. **(A)** SVM based model performance for 5-subject and 7-subject experiment, **(B)** confusion matrix for 7-subject experiment, **(C)** confusion matrix for 5-subject experiment.

To investigate the impact of challenging subjects on model performance, subjects Sb106 and Sb381 were excluded from the analysis. Following their exclusion, a notable improvement in identification accuracy was observed across all evaluated metrics. Figure 10 illustrates the effect of reducing the number of less identifiable subjects when employing the SVM-based classification model for resting state stimuli. The bar plot presents a comparative analysis of performance metrics — including accuracy (ACC), F1-score, AUC, recall, precision, and specificity — accompanied by standard deviation values across the 5 fold performance, while the confusion matrices provide a detailed visualization of the classification outcomes for both experimental configurations.

### 5.1 Comparison with the state of the art

A common methodological flaw in EEG-based biometric studies involves training and testing models on data obtained from the same or mixed EEG recording sessions, which can lead to overly optimistic accuracy estimates (Arnau-González et al., 2021). To ensure a fair evaluation of biometric performance, longitudinal analysis—highlighted by Takashi et al. (2017) and adopted in the present study—is essential. This issue was also noted by Renata et al. (2023) who reported a 20% decline in accuracy when cross-session data were used. Similarly, Milan et al. observed a 10% reduction in accuracy when training and testing were performed on data from different sessions (Milan and Jakub, 2012). Using the publicly available SEED dataset, cross-session classification achieved 79.34% accuracy with an SVM classifier (Pablo et al., 2021). Other studies employing various pattern classification methods for subject identification have reported accuracy scores ranging from 82 to 97% (Armstrong et al., 2015).

Rig et al. (2016) developed an event-related potential (ERP)-based framework that achieved 95% accuracy when trained and tested across separate sessions. Ozan et al. (2019) combined deep learning with quadratic discriminant analysis (QDA) on a dataset of 10 subjects, attaining 72% accuracy. Milan and Jakub (2012) utilized autoregressive (AR) features with distance-based classification for nine subjects, reporting an accuracy of 77%. Lyakhov et al. (2021) achieved an exceptionally low equal error rate (EER) of 2% using AR features and hidden Markov models (HMM) with data from 45 subjects. Pablo et al. (2021) integrated AR, fractal complexity coefficients, and power spectral density features with multiple classifiers—such as SVM, KNN, AdaBoost, and MLP—achieving up to 73% accuracy with 15 subjects.

The authors recently proposed an autoencoder-based frameworks for subject identification using EEG data cubes (Oztemel and Soysal, 2025). In that study, features were extracted by means of a classical autoencoder model; the highest AUC score of 90.49% and ACC score of 59.09% were achieved in identification out of 7 subjects. In this study, an attention mechanism was integrated into the autoencoder architecture to emphasize the effective patterns in the resulting latent feature representation. A maximum AUC of 90.99% and ACC of 62.90% in identification tasks were observed. Furthermore, experiments conducted on the dataset obtained from five participants, after excluding less identifiable subject, confirmed that the attention-based model achieved a significant improvement compared to the basic autoencoder-based identification scores reported in Oztemel and Soysal (2025). The performance scores for autoencoder with attention increased to 96.82% from 95.19% and to 82.54% from 79.43% for AUC and ACC, respectively.

### 5.2 Limitation and future work

It is worth highlighting that this study serves as an early and pioneering effort in its methodological approach. The proposed attention-based framework has been shown to enhance the effectiveness of EEG feature extraction and provides promising results for subject identification using 3D representations of EEG data. Nevertheless, the dataset used in this work comprises a limited number of participants and relatively short intervals between recording sessions. This limitation restricts the diversity of inter-subject variability captured by the model and may affect its ability to generalize across larger and more heterogeneous populations.

In addition, the constrained sample size may impact the robustness of the proposed biometric system, particularly in real-world scenarios where intra-subject variability is significantly higher. As a result, the reported performance should be interpreted as preliminary.

Sufficient training data may present challenges where EEG data is limited. Model performance decreases as the number of subjects enhanced which is an inherent struggle in biometric studies. Adequate data can be a solution for developing robust EEG based identification models. Additionally, variations in EEG acquisition between sessions—often unavoidable—may affect the model’s performance. The framework’s generalizability across diverse EEG systems and recording settings remains to be validated. Additionally, artifacts arising from subject behavior, such as eye blinks and muscle movements, may influence the model’s performance. Future research should systematically examine artifact removal methods to strengthen the robustness and adaptability of the proposed framework. Moreover, a comprehensive usability assessment framework will be employed to evaluate the system by considering multiple user-related factors from a user-centered perspective, equal error rate and receiver operating characteristics curve.

To address these limitations, future research will focus on validating the proposed framework using larger-scale and publicly available EEG datasets, incorporating a greater number of participants and longer longitudinal recording intervals. Also, statistical analysis such as ANOVA will be applied to rigorously assess the significance of performance differences across models and frequency bands. Such evaluations will enable a more comprehensive assessment of the model’s generalizability, stability, and robustness in practical biometric applications.

## 6 Conclusion

In this study, attention mechanism–based autoencoder models were developed for EEG feature extraction. The extracted features were then utilized for personal identification using KNN, RF, ANN, and SVM classifiers. These models were evaluated across eight different EEG frequency bands and three stimulus types, including resting-state, auditory, and cognitive tasks. Among all configurations, the SVM model applied to the Gamma band of resting-state EEG recordings achieved the highest performance, reaching an AUC of 90.99% and ACC of 62.90% when the seven-subject were identified. Subject identifiability was assessed using pairwise comparisons between all subject pairs, yielding accuracy scores at AUC of 99.73% and ACC of 97.62%. After excluding two subjects with lower identifiability, the five-subject experiment achieved an AUC of 96.82% and ACC of 82.54%. Overall, these findings demonstrate that the proposed attention-based autoencoder framework provides a promising foundation for advancing EEG-based personal identification systems.

## Data Availability

The data analyzed in this study is subject to the following licenses/restrictions: data will be shared upon request. Requests to access these datasets should be directed to omer.soysal@selu.edu.
